# Trait Mindfulness Is Associated With the Self-Similarity of Heart Rate Variability

**DOI:** 10.3389/fpsyg.2019.00314

**Published:** 2019-02-28

**Authors:** Shasha Sun, Chuanlin Hu, Junhao Pan, Chengyi Liu, Miner Huang

**Affiliations:** ^1^Department of Psychology, Sun Yat-sen University, Guangzhou, China; ^2^School of Physical Education and Sports Science, South China Normal University, Guangzhou, China

**Keywords:** trait mindfulness, self-similarity, heart rate variability (HRV), crosstalking, autonomic nervous system (ANS)

## Abstract

Previous studies have linked trait mindfulness with better self-regulation and adaptation. Heart rate variability (HRV) is a good physiological indicator of the capacity for self-regulation and adaptation. The present study explored the relationship between trait mindfulness and HRV from the viewpoint of crosstalking between different HRV parameter pairs, which would reflect the dynamic interactions between each pair of HRV parameters in different processes. We measured the trait mindfulness of seventy-four undergraduate students and recorded nine HRV parameters during the following four consecutive experimental phases: (1) calming phase, (2) mental arithmetic task phase, (3) recovery phase, and (4) mindfulness practice phase. The relationship between trait mindfulness and HRV was explored at the following three levels: (1) the absolute level, i.e., HRV parameters in four different states, (2) the difference-change level, i.e., differences in HRV parameters between different states, and (3) the crosstalking level, i.e., self-similarity of crosstalking HRV parameter pairs. The results supported the following hypothesis: trait mindfulness, as measured by the Mindful Attention Awareness Scale (MAAS), was significantly and positively correlated with the self-similarity of crosstalking HRV parameter pairs but was not significantly correlated with the HRV parameters at the difference-change and absolute levels. These findings indicate that as trait mindfulness increases, the ability to maintain ANS function homeostasis improves.

**HIGHLIGHTS**

- Trait mindfulness is associated with better self-regulation and adaptation.
- Heart rate variability (HRV) is a good physiological indicator of the capacity for self-regulation and adaptation.
- Trait mindfulness is significantly correlated with self-similarity of crosstalking HRV parameter pairs but not with the HRV parameters at the difference-change or absolute levels.

## Introduction

The way individuals respond to the constantly changing environment has profound implications for physical and psychological health (Cacioppo, 1998; Schetter and Dolbier, 2011). Previous studies have linked the psychological construct of trait mindfulness with better self-regulation and adaptation (e.g., McCraty and Childre, 2010; Keng et al., 2011), while heart rate variability (HRV) is considered a good physiological indicator of the capacity for self-regulation and adaptation (e.g., Geisler and Kubiak, 2009; Reynard et al., 2011). Therefore, the relationships between trait mindfulness and HRV are of considerable interest. However, existing studies investigating such relationships have revealed mixed results. While some studies have observed significant associations between trait mindfulness and HRV (e.g., Garland, 2011; Fogarty et al., 2015), other studies did not observe significant relationships (e.g., Soer et al., 2015; Jäger, 2016). To further clarify this question, we explore the relationship between trait mindfulness and HRV at the following three levels to address key questions regarding methodological and theoretical significance: (1) the absolute level, i.e., HRV parameters in four different states; (2) the difference-change level, i.e., differences in HRV parameters between different states; and (3) the crosstalking level, i.e., self-similarity of crosstalking HRV parameter pairs.

### Trait Mindfulness and Adaptation

Mindfulness, which is defined as intentional present-moment non-judgmental awareness, fosters a healthy response by allowing people to better regulate their subjective and physiological responses to the changing environment (Kabat-Zinn, 2005). Mindfulness can be considered a personality trait (Brown and Ryan, 2003) and has been shown to be strongly associated with flexible responses to stimuli, increased subjective well-being, and reduced psychological and physiological symptoms (Epel et al., 2009; Keng et al., 2011; Silberstein et al., 2012). People with high levels of trait mindfulness have been confirmed to exhibit a propensity to attend to their present-moment experience as a means of becoming aware of their automatic reactions and, thus, remain non-reactive in the face of distressing thoughts, emotions, and somatic sensations (Garland, 2007; Garland et al., 2010; McCraty and Childre, 2010).

### Heart Rate Variability (HRV) and Adaptation

Some researchers have noted that the ability to respond to ongoing internal and external circumstances in healthy and effective ways greatly depends on the synchronization, sensitivity, and stability of our biological systems (McCraty and Childre, 2010). HRV, which refers to the change in the time intervals between consecutive heart beats, is a widely used index of the synergistic actions of the sympathetic nervous system (SNS) and the parasympathetic nervous system (PNS) in the autonomic nervous system (ANS) (Berntson et al., 1997; Cacioppo et al., 2007; Karim et al., 2011). HRV can be quantified in various ways that can be broadly classified into time and frequency-domain measures (Berntson et al., 1997). The two commonly used time-domain measures are the standard deviation of all normal to normal intervals (SDNN) and the root mean square of successive differences between normal heartbeats (RMSSD). Specifically, SDNN reflects the ebb and flow of all factors that contribute to HRV (Shaffer et al., 2014), while RMSSD reflects the beat-to-beat variance in heart rate (HR) and is the most common time-domain measure used to estimate the vagally mediated changes in HRV. The frequency-domain measures can be further categorized, and the total power (TP) is divided into very low-, low- and high-frequency components. At the level of the sinoatrial (SA) node, very low-frequency HRV (VLF-HRV; 0.0033–0.04 Hz) indicates healthy function, and an increase in the resting VLF-HRV and/or a shift in frequency can reflect efferent sympathetic activity. Low-frequency HRV (LF-HRV; 0.04–0.15 Hz) captures sympathetic and parasympathetic activity, while high-frequency HRV (HF-HRV; 0.15–0.40 Hz) captures inhibitory parasympathetic (i.e., vagal) input. RMSSD has been shown to be positively correlated with HF-HRV (Kleiger et al., 2005). Coherence is another important frequency-domain measure that may reflect frequency entrainment among respiration, blood pressure, and heart rhythms; synchronization among systems; the stability of a single wave form, such as respiration or HRV patterns; and system resonance (McCraty and Childre, 2010; Shaffer et al., 2014; McCraty and Shaffer, 2015).

Studies have increasingly linked HRV to self-regulation and adaptability (e.g., Holzman and Bridgett, 2017; Shaffer and Ginsberg, 2017). Higher levels of HRV and context-appropriate changes in HRV are associated with enhanced attention regulation abilities (Appelhans and Luecken, 2006) and better behavioral regulation (Segerstrom and Nes, 2007). Reduced HRV across situations may indicate a lack of autonomic flexibility, which is necessary for healthy functioning (Friedman, 2007; Beauchaine and Thayer, 2015).

### Trait Mindfulness and HRV

Based on the above studies, we could conclude that both trait mindfulness (i.e., a psychological variable) and HRV (i.e., a physiological indicator of ANS function) can be used to reflect an organism’s flexibility and ability to adapt to constantly changing internal and external environments. However, research examining the relationship between trait mindfulness and HRV is limited. Among the few existing studies, Garland (2011) found that higher trait mindfulness was significantly associated with greater HF-HRV recovery from stress-primed alcohol cues among 58 alcohol-dependent inpatients. Fogarty et al. (2015) found that participants who were more mindful showed greater HF-HRV in an emotional writing task, followed by superior recovery, indicating that trait mindfulness may facilitate more adaptive responses under stress. Mankus et al. (2013) examined the relationship between trait mindfulness and HRV in individuals with generalized anxiety (GA) symptoms. These authors found that trait mindfulness was positively associated with HRV only in the high-GA group and not in the low-GA group.

The studies mentioned above revealed a significant correlation between trait mindfulness and HRV, but the results are not entirely consistent. Jäger (2016) measured trait mindfulness in 106 undergraduate students and recorded their HRV in a resting state. The results showed no significant association between trait mindfulness and HRV (SDNN, LF-HRV and VLF-HRV). Soer et al. (2015) applied heart coherence training in a study involving 10 patients with chronic musculoskeletal pain and 15 healthy subjects. Compared to the baseline levels, both trait mindfulness and the coherence score increased after three sessions of training, but no significant correlation was observed between the change in trait mindfulness and the change in the coherence score.

There are several limitations to the previous studies. Although many HRV parameters exist, most previous studies reported only one or two statistically significant results, which may be due to publication bias. However, organisms respond to the environment holistically instead of depending on only one or two functions represented by specific measures. Therefore, a methodology that depends on only one or two specific measures could fail to collect valuable information. Another reason for reporting only one or two HRV parameters is that the implications of some HRV parameters, such as HF-HRV and RMSSD, are clearer than those of other HRV parameters, such as LF-HRV. Moreover, limited by traditional methods, most previous studies calculated the correlations between trait mindfulness and the HRV parameters individually; this approach cannot reflect the dynamic interactions between each pair of parameters. In addition, according to Whitehead et al. (1978), reality is formed by processes rather than material objects, and the best definition of a process is based on its relationships to other processes. Fechner (2003) stated that the just-noticeable difference between two stimuli is proportional to the magnitude of the stimuli. Thus, many sensory systems respond to a stimulus proportionally to the fold change between the stimulus and background. Therefore, it is possible that the relationship between trait mindfulness and HRV is more likely to exist at the crosstalking level between HRV parameter pairs than at the difference-change or absolute levels.

### Fractal Scaling and Self-Similarity Algorithm

We introduce a new algorithm called the self-similarity algorithm (Liu C.Y. et al., 2017; Liu T.C. et al., 2017; Liu et al., 2018) to address the question of whether the relationship between trait mindfulness and HRV exists at the crosstalking, difference-change or absolute level. In the following text, we first introduce the concept of self-similarity and fractal scaling, and then introduce the self-similarity algorithm in detail.

**FIGURE 1:**
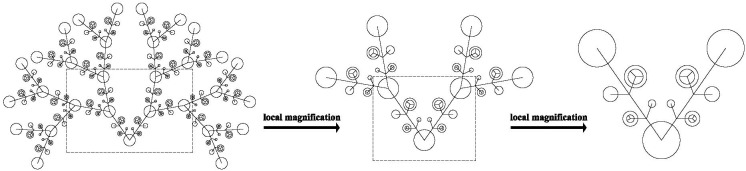
Diagram of self-similarity. Drawn by Mian Tang, South China Normal University.

In physical and life sciences, fractal scaling is considered to be a fundamental property of non-linear, complex dynamical systems (Mandelbrot, 1998). Self-similarity (see Figure 1) is derived from the fractal literature as follows: a pattern is self-similar if it does not vary with spatial or temporal scales (Mandelbrot, 1983). Self-similar phenomena are widespread in nature. For example, as demonstrated by Figure 1, the local/branches of a tree, i.e., small scale structures, are similar but not necessarily identical to the whole tree, i.e., the larger scale form. This phenomenon is spatially self-similar. Temporal self-similarity means that variability is statistically similar across different processes and multiple time scales (seconds, minutes, etc.). All of the points in a fractal process possesses a “memory” of preceding points of the process, and therefore all of the points in the process are embedded in the historical context of the system (Diniz et al., 2011). The fractal process indicates a dynamic balance between order and chaos, or flexibilities and homeostasis (Wijnants et al., 2012; Sun et al., 2018). Temporal self-similarity could manifest in processes through algorithms such as detrended fluctuation analysis (DFA) (Peng et al., 1995) and our self-similarity algorithm (Liu C.Y. et al., 2017; Liu T.C. et al., 2017; Liu et al., 2018). It is found that the temporal structures of circadian rhythms such as heartbeats and psychological experience such as emotions demonstrated fractal, self-similar natures (e.g., Goldberger, 1991; Ruiz-Padial and Ibáñez-Molina, 2018). Moreover, the temporal structures of subtle body movement fluctuations and state self-esteem variability also exhibited self-similarity (D’Mello et al., 2012; De Ruiter et al., 2015). The following is the self-similarity algorithm.

The power law between parameters *x* and *y* was produced with a self-similarity condition:

(1) $$y=ax^{1}$$

where *a* and *l* are constants (Harte et al., 1999; Liu C.Y. et al., 2017; Liu T.C. et al., 2017; West, 2017; Liu et al., 2018). The power law and experimental investigations have always been linked in fractal physics, physiology, and psychology in the spatial or temporal domains or its Fourier transformation domain; thus, the power law, Eq. (1), exactly holds true (Mandelbrot, 1983; West, 2017; Sen and McGill, 2018). However, the power law, Eq. (1), may not hold true, especially in life science. Our self-similar algorithm (Liu C.Y. et al., 2017; Liu T.C. et al., 2017; Liu et al., 2018) introduced the self-similar exponent (SSE) and quantitative difference (QD) so that we can study the situation where the power law, Eq. (1), approximately holds according to QD. The data topology is quantitatively represented with the following process logarithm and its SSE:

(2) $$lp_{z}(i,j)=\log_{\tau }\;z_{i}/z_{j},\;i\neq \;j,\;i,\;j=1,\;2,\;\ldots ,\;N,\;z=x\;\mathrm{or}\;y,\;\tau =\left(\sqrt{5}-1\right)/2$$

(3) $$SSE(i,j)=lp_{y}\;(i,\;j)/lp_{x}\;(i,\;j),\;i\neq \;j,\;i,\;j=1,\;2,\;\ldots ,\;N$$

The SSE is exactly conserved if the power law, Eq. (1), exactly holds true

(4) SSE(i,j)=l, i≠ j, i, j=1, 2, …, N

However, the SSE series are different from one another if the power law, Eq. (1), do not holds true. SSE can be studied as a process parameter to represent the crosstalking between discrete data sets {*x*_i_, *i* = 1, 2, …, *N*} and {*y*_i_, *i* = 1, 2, …, *N*}. The absolute value of *lp*_*z*_ (*i, j*) is defined as the QD between the two values of parameter *z* (*z* = *x* or *y*), but the QD between two different SSEs is calculated as follows:

(5) $$QD_{SSE}=|0.5[SSE(i,j)+SSE(k,m)]\;\ln\;SSE(i,j)/SSE(k,m)|,\;i\neq \;j,k\neq \;m,\;\;i,\;j,\;k,\;m=1,\;2,\;\ldots ,\;N$$

The crosstalking between the two data sets is approximately self-similar if the QD between any two SSEs in the data set {*SSE* (*i, j*), *i* ≠ *j, i, j* = 1, 2, …, *N*} is not significant, which is also approximately called SSE conservation.

The QD thresholds, i.e., (α, β), are used to determine whether the SSEs are conservative. The QD may be understood as negative feedback (Brown et al., 2007; Liu et al., 2012, 2014, 2018; Liu C.Y. et al., 2017; Liu T.C. et al., 2017). Function-specific homeostasis (FSH) refers to the biological negative feedback response that maintains the specific conditions of a function allowing that function to be performed perfectly. A function in its FSH is called a normal function, while a function far from its FSH is regarded as a dysfunctional function. A normal function is capable of resisting disturbances under its threshold such that QD < α for the disturbance-induced difference. A dysfunctional function sensitively depends on a disturbance such that the QD is significant if α ≤ QD < β and extremely significant if QD ≥ β for the disturbance-induced difference. The QD thresholds at the level of organs or tissues are approximately (0.27, 0.47) (Liu et al., 2012, 2014, 2018; Liu C.Y. et al., 2017; Liu T.C. et al., 2017). The function of HRV is at this level; thus, when all of the QD_SSE_ values of the two HRV parameters in Eqs. (3) and (5) in the different subprocesses of a process are smaller than 0.47, the crosstalking between the two parameters is considered to remain self-similar in the process.

### The Present Study

Heart rate variability reflects flexible adaptation and the limit of flexibility, i.e., homeostasis, which is equally important to one’s health and well-being. Our self-similarity algorithm (Liu C.Y. et al., 2017; Liu T.C. et al., 2017; Liu et al., 2018) can be utilized to address the homeostasis of one’s functions in different processes. According to previous studies, the experimental structure, including a baseline, task, and recovery process, is commonly recommended for measuring HRV (Berna et al., 2014; Holzman and Bridgett, 2017; Laborde et al., 2017). In addition, mental arithmetic can produce acute mental stress (Visnovcova et al., 2014); thus, we established four conditions (a calming phase, a mental arithmetic task phase, a recovery phase, and a mindfulness practice phase) to induce different physical and psychological states and to examine the self-similarity of HRV between two different processes.

Mandelbrot (1983) pointed out, in physical and life sciences, fractal scaling is considered to be a fundamental property of complex dynamical systems. There are two main differences between our self-similarity algorithm and DFA. One lies in that, DFA and other finite-variance scaling methods tacitly admitting the power law [refer to Eq. (1)] exactly hold true; but we believe that the power law can only approximately holds true, especially in life sciences. The other one lies in that, DFA is utilized to analyze the variability and invariance of one single parameter varying with time, and our self-similarity algorithm is utilized to analyze the crosstalking between parameter pairs. As mentioned above, there exist many HRV parameters, but until now, rare studies explore ANS function from the viewpoint of the dynamic interactions between each pair of HRV parameters. The present study is to explore ANS function using self-similarity algorithm, and then the relationship between trait mindfulness and HRV self-similarity.

In summary, the aim of the present study was to explore the relationship between trait mindfulness and HRV at the absolute level and the difference-change level using a traditional method and at the crosstalking level using our self-similarity algorithm (Liu T.C. et al., 2017; Liu C.Y. et al., 2017; Liu et al., 2018). As a complex system, the crosstalking of HRV parameter pairs would manifest a self-similarity process as well as FSH. According to the notion that mind-body coupling is a self-organizing process (Kello et al., 2007), and trait mindfulness helps self-organizing, we hypothesized that the relationship between trait mindfulness and HRV exists most likely at the crosstalking level rather than at the absolute or difference-change level.

## Materials and Methods

### Participants

Seventy-four undergraduate students (15 males and 59 females) participated in this study. The participants were recruited from a WeChat^1^ Group. The mean age of the participants in the sample was 18.28 ± 1.08. None of the participants had meditation experience, and they were not familiar with mindfulness. All participants provided written informed consent according to the study protocol, which was approved by the Ethics Committee of the Department of Psychology, Sun Yat-sen University.

### Procedures

The participants were oriented to the physiological recording equipment and briefed regarding the experimental procedure. The participants signed the informed consent form and completed the questionnaires. The questionnaires included information about the demographic variables and mindfulness self-ratings. Then, the participants were subjected to all four phases of the experiment, which included a 3-min calming phase, a 5-min mental arithmetic task, a 3-min recovery phase, and a 5.4-min breathing space exercise. The HRV parameters were recorded during the four phases.

### Materials

#### Phases 1 and 3

Thirty-six different scenery pictures were used (18 pictures per phase). PowerPoint was used to present the pictures (10 s each). The participants viewed the pictures to calm themselves in phase 1 and to recover from the mental arithmetic task in phase 3.

#### Phase 2: Mental Arithmetic Task

**FIGURE 2:**
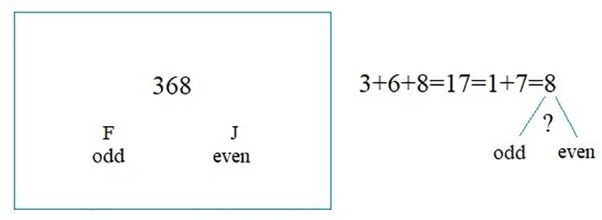
Mental arithmetic task.

Mental arithmetic tasks (see Figure 2 for an example) represent a standard stressor with a moderate intensity used in physiology studies to detect changes in ANS function (Schneider et al., 2003). The participants calculated three-digit numbers displayed on the screen into one-digit numbers. Then, the participants indicated whether the final result was even or odd by pushing keys (F for odd, J for even, see Visnovcova et al., 2014). E-prime 2.0 was used to program and display the numbers. Seven numbers were used as practice, and 60 different numbers were used in the formal experiment. Each number was displayed for a maximum of 3,500 ms. The reaction times and responses were recorded.

#### Phase 4: Breathing Space Exercise

A 5.4-min Chinese version of the breathing space exercise audio produced by Dr. HuiQi Tong was used to induce a mindfulness state. This exercise is a core exercise of mindfulness-based cognitive therapy (MBCT) and is described as “mini-meditation.” The three steps of the breathing space exercise are awareness (currently occurring internal experiences), focus (breathing), and expand (the body as a whole) (Segal et al., 2002).

### Measures

#### Heart Rate Variability

Heart rate variability was continuously recorded with the emWave Pro Plus system (HeartMath LLC., Boulder Creek, CA, United States). The software version of the system was 3.6.0.9625, and the software provided a sampling rate of 370 Hz and automatic pulse wave detection and calibration. The hardware of the system was an infrared pulse plethysmograph (ppg) ear sensor. The operation system used was Microsoft Windows 10.

The HRV parameters included SDNN, RMSSD, TP, VLF-HRV, LF-HRV, HF-HRV, the LF/HF ratio, and coherence. Based on the results presented by the emWave Pro Plus system, the mean HR was classified into the time-domain measure of HRV. Therefore, the following HRV parameters were calculated and are reported in the present study: HR, SDNN, RMSSD, TP, VLF-HRV, LF-HRV, HF-HRV, the LF/HF ratio, and coherence.

#### Questionnaire

The Mindful Attention Awareness Scale (MAAS) (Brown and Ryan, 2003) is the most widely used trait mindfulness scale (Medvedev et al., 2016). This scale is a 15-item (e.g., “I could be experiencing some emotion and not be conscious of it until later”) measure of individual differences in how frequently individuals are in the mindful states over time using a 6-point Likert scale ranging from 1 (almost always) to 6 (almost never). High scores reflect high levels of trait mindfulness. The total MAAS score of the 74 participants was 56.01 ± 9.68. The internal consistency in this sample was acceptable, and Cronbach’s alpha was 0.811.

### Statistical Analysis

#### Data Inclusion Criteria

The accuracy of the 74 participants on the mental arithmetic task ranged from 40 to 97%. We divided the participants into low- and high-accuracy groups according to the median of their overall scores, i.e., 75.83%. There were 37 participants whose accuracy was lower than 75.8%; these participants were included in the low-accuracy group. The other 37 participants were included in the high-accuracy group. The reaction times of the low- and high-accuracy groups were 2450.04 ± 204.08 ms and 2209.73 ± 250.76 ms, respectively; and the accuracies of the low- and high-accuracy groups were 0.66 ± 0.09 and 0.84 ± 0.05, respectively. An independent-samples *t*-test showed that the reaction time of the high-accuracy group was significantly shorter than that of the low-accuracy group, *t*(72) = -10.49, *p* < 0.001, Cohen’s *d* = 1.05; additionally, the accuracy of the high-accuracy group was significantly higher than that of the low-accuracy group, *t*(72) = 4.52, *p* < 0.001, Cohen’s *d* = 2.44. The scores on the MAAS and HRV parameters in the four phases did not significantly differ between the two groups, *p*s > 0.05. The physiological reaction responses represented by the HRV parameters were the same between the two groups. These results revealed that the poor performance of the low-accuracy group in the mental arithmetic task was possibly caused by their pre-existent weakness in arithmetic tasks and not by a lack of seriousness in task performance. In addition, the two groups’ MAAS scores revealed that they were homogeneous in trait mindfulness. Therefore, the data of all 74 participants were used in the following analysis.

#### Calculation of Self-Similarity of HRV Parameters

The four phases of the present study were as follows:

Phase 1: a 3-min calming phase,

Phase 2: a 5-min mental arithmetic task,

Phase 3: a 3-min recovery phase, and

Phase 4: a 5.4-min breathing space exercise.

As shown in Table 2, HR, SDNN, RMSSD, TP, VLF-HRV, LF-HRV, and HF-HRV were the highest points, while the LF/HF ratio and coherence were the lowest points in the mental arithmetic task relative to the other three phases. Therefore, phase 2 was selected as the starting point according to Liu T.C. et al., 2017; Liu C.Y. et al., 2017. We calculated the self-similarity of the HRV parameters in subprocesses from phases 2 to 3 and phases 2 to 4. Excel 2016 was used to complete the calculations. The steps were as follows:

(1) Using Eq. (6), we obtained two process logarithms (PLs), i.e., the PL of the subprocesses from phases 2 to 3 and the PL of the subprocesses from phases 2 to 4 for *x* = LF-HRV and *y* = HF-HRV, respectively.
  (6) $$lp_{z}\;(2,\;i)=\log_{\tau }\;z_{2}/z_{i},\;i=3,\;4,\;z=x\;\mathrm{or}\;y,\;\tau =\left(\sqrt{5}-1\right)/2$$
(2) Using Eq. (7), we obtained two SSEs.
  (7) $$SSE\;(2,\;i)\;=lp_{y}\;(2,\;i)/lp_{x}\;(2,\;i),\;i=3,\;4,$$
(3) Using Eq. (8), we obtained one QD.
  (8) $$QD_{SSE}=|0.5(SSE\;(2,\;3)\;+SSE\;(2,\;4))\;\ln\;SSE\;(2,\;3)/SSE\;(2,\;4)|$$
(4) Steps 1–3 were repeated to complete the calculation of the self-similarity of all other pairs of HRV parameters.
(5) The QD thresholds at the level of organs or tissues are approximately (0.27, 0.47) (Liu et al., 2012, 2014, 2018; Liu C.Y. et al., 2017; Liu T.C. et al., 2017). As shown in the “Introduction” section, QD_SSE_ < 0.47 indicates that the pair of HRV parameters remains self-similar, while QD_SSE_ ≥ 0.47 indicates that the pair of HRV parameters does not remain self-similar. Nine parameters of HRV are used in the present study; thus, each participant should have 36 QDs as calculated by the formula C_9_^2^ = (9 × 8)/2 = 36. Finally, the self-similarity of HRV is represented by the number of QD_SSE_ which is smaller than 0.47.

## Results

### Means and Standard Deviations of HRV Parameters in the Four Phases

Linear mixed effects models controlling for subject-specific random intercept were used to analyze the effects of the four phases on the nine HRV parameters. The effects of the phases and gender were considered as fixed effects, and the unstructured repeated covariance type was selected.

The results revealed that regarding the parameters HR, SDNN, RMSSD, TP, VLF-HRV, LF-HRV, HF-HRV, and LF/HF ratio, gender showed a non-significant effect, *p*s > 0.05; for the parameter coherence only, gender showed a significant effect, *F*(1,72) = 4.62, *p* = 0.04. Regarding the parameters HR, VLF-HRV, LF-HRV, HF-HRV, LF/HF ratio and coherence, the phases showed significant effects, *p*s < 0.01; regarding the parameters SDNN and TP, the phases showed marginal significant effects, *p*s = 0.08; regarding the parameter RMSSD, the phases showed a non-significant effect, *p* > 0.10. Table 1 lists the *F-* and *p*-values from the linear-mixed effects for the effects of gender and the phases on model parameters.

**Table 1:** *F-* and *p*-values from the linear-mixed effects for the effects of gender and the phases on model parameters.

	Fixed effects			
	
	Gender (*df* = 1,72)		Phases (*df* = 3,73)	
		
Parameters	*F*-value	*p*-value	*F*-value	*p*-value
HR	0.03	0.86	25.18	<0.01
SDNN	1.34	0.25	2.34	0.08
RMSSD	0.04	0.84	0.32	0.81
TP	3.10	0.08	13.09	<0.01
VLF-HRV	2.27	0.14	5.50	0.01
LF-HRV	2.35	0.13	25.05	<0.01
HF-HRV	0.05	0.83	4.95	<0.01
LF/HF ratio	2.59	0.11	19.81	<0.01
coherence	4.62	0.04	36.85	<0.01


*HR, heart rate; SDNN, standard deviation of the normal to normal (NN) intervals; RMSSD, root mean square of successive differences between normal heartbeats; TP, total power; VLF, very-low-frequency; LF, low-frequency; HF, high-frequency; HRV, heart rate variability. The same is true below.*

Table 2 lists the pairwise comparisons of the nine HRV parameters in the four phases.

**Table 2:** Means and standard deviations of the HRV parameters in the four phases (*n* = 74).

HRV parameters		Phase 1 Calming phase *M* ±*SD*	Phase 2 Mental arithmetic task *M* ±*SD*	Phase 3 Recovery phase *M* ±*SD*	Phase 4 Mindfulness practice *M* ±*SD*
Time-domain measures	HR	80.29 ± 10.63c	84.98 ± 11.39a	79.53 ± 9.50c	81.49 ± 9.17b
	SDNN	54.17 ± 17.86	52.04 ± 15.74b	53.68 ± 17.07b	57.37 ± 18.13a
	RMSSD	46.79 ± 17.19	45.79 ± 17.74	50.45 ± 54.07	46.27 ± 38.45
Frequency-domain measures	TP	860.32 ± 618.67b	681.35 ± 436.44c	989.28 ± 1084.81b	1257.89 ± 887.70a
	VLF-HRV	287.85 ± 286.88b	235.14 ± 223.17c	380.36 ± 462.61a	327.69 ± 250.40b
	LF-HRV	310.68 ± 349.50b	175.33 ± 141.75c	316.44 ± 258.78b	624.08 ± 571.46a
	HF-HRV	261.78 ± 251.17a	208.79 ± 172.11b	214.74 ± 195.68b	257.32 ± 238.82a
	LF/HF ratio	1.65 ± 2.00b	1.11 ± 0.80c	2.01 ± 1.50b	3.29 ± 2.83a
	Coherence	37.85 ± 9.51b	32.16 ± 5.79c	37.70 ± 7.59b	43.16 ± 8.42a


*In each row, a, b, and c after *M* ±*SD* indicate significant differences between each two means in different phases based on Fisher’s LSD *post hoc* paired comparisons.*

### Correlations Between the MAAS Score and HRV Parameters at the Absolute Level

Bivariate correlations were carried out between the MAAS score and HRV parameters at the absolute level in the four phases. As shown in Table 3, no significant correlations emerged between the MAAS score measures in any phase.

**Table 3:** Spearman’s correlation coefficient *ρ* between the MAAS score and HRV parameters at the absolute level (*n* = 74).

Measures	Calming	Mental arithmetic task	Recovery	Mindfulness practice
HR	-0.095	-0.124	-0.183	-0.194
SDNN	0.117	0.107	0.047	0.074
RMSSD	-0.043	-0.010	-0.028	-0.100
Total Power	0.161	0.165	0.090	0.058
VLF-HRV	0.225	0.148	0.143	0.091
LF-HRV	0.065	0.086	-0.132	-0.019
HF-HRV	0.010	0.122	0.111	-0.019
LF/HF ratio	0.026	-0.127	-0.172	-0.034
Coherence	0.018	-0.098	0.001	-0.019


*MAAS score = 56.01 ± 9.68.*

### Correlations Between the MAAS Score and HRV Parameters at the Difference-Change Level

Bivariate correlations were carried out between the MAAS scores and HRV parameters at the difference-change level, i.e., phase 2–phase 1 indicates mental arithmetic task reactivity, and phase 4–phase 3 indicates mindfulness practice reactivity. The results revealed that the MAAS scores were not significantly correlated with the HRV parameters in phase 2–phase 1 or phase 4–phase 3 (see Table 4).

**Table 4:** Spearman’s correlation coefficient *ρ* between the MAAS score and difference-change of the HRV parameters in different processes.

Phase 2–Phase 1		Phase 4–Phase 3	
HR2–HR1	-0.103	HR4–HR3	0.017
SDNN2–SDNN1	-0.045	SDNN4–SDNN3	-0.026
RMSSD2–RMSSD1	0.016	RMSSD4–RMSSD3	-0.067
TP2–TP1	-0.026	TP4–TP3	0.013
VLF2–VLF1	-0.096	VLF4–VLF3	-0.061
LF2–LF1	-0.051	LF4–LF3	0.072
HF2–HF1	0.093	HF4–HF3	-0.077
Ratio 2–Ratio 1	-0.107	Ratio 4–Ratio 3	0.067
Coherence 2–coherence 1	-0.108	Coherence 4–coherence 3	-0.086


### Correlations Between the MAAS Score and HRV Parameters at the Crosstalking Level

**FIGURE 3:**
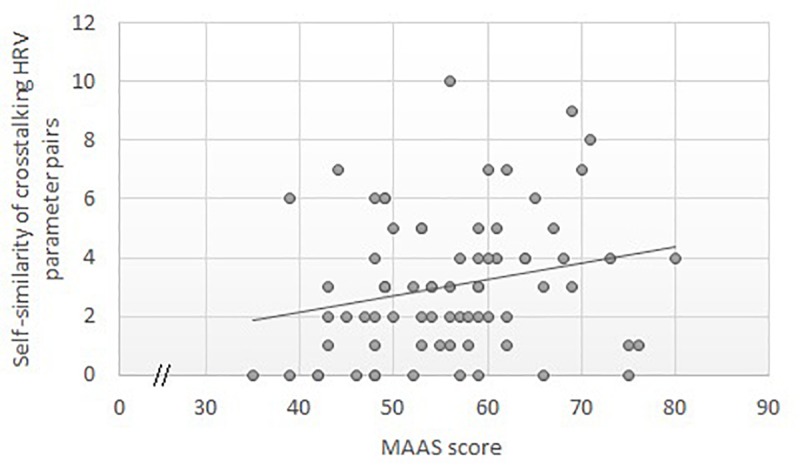
Scatter plot of the correlations between the MAAS score and self-similarity of the HRV parameters.

In the present study, the mean number of pairs of HRV parameters whose QD < 0.47 was 3.05 ± 2.37, ranging from 0 to 10. A significant correlation emerged between the MAAS scores and the number of pairs of HRV parameters, i.e., the self-similarity of the HRV parameters in different processes; Spearman’s *ρ* = 0.26, *p* = 0.023. These results indicate that the relationship between the MAAS score and HRV emerged at the crosstalking level (see Figure 3). Thus, the greater the crosstalking HRV parameter pair in self-similarity is, the higher the MAAS score.

## Discussion

The current study examined whether trait mindfulness is associated with changes in physiological markers of self-regulation under four different conditions, i.e., a calming phase, a mental arithmetic task, a recovery phase, and mindfulness practice. The calming phase was considered the baseline, and the mental arithmetic task induced a stressful physiological reaction, which was followed by physiological downregulation during the recovery phase. The mindfulness practice phase was used to determine whether trait mindfulness is associated with HRV parameters during mindfulness practice. In addition, it is necessary to calculate the self-similarity of the HRV parameters. Based on the traditional method, we examined the relationships between trait mindfulness and the HRV parameters at the absolute level and the difference-change level. We also examined the relationships between trait mindfulness and the self-similarity of the HRV parameters at the crosstalking level in accordance with Liu C.Y. et al., 2017; Liu T.C. et al., 2017; Liu et al., 2018. Whether the power law, Eq. (1), holds true is the main point of the difference of our self-similar algorithm from the fractal physics. For the fractal physics, the power law exactly holds true so that the SSE is exactly conserved and the self-similarity exactly holds true. However, the power law may not holds true, especially in life science. Our self-similar algorithm then introduced the SSE and QD so that we can study the situation where the power law does not exactly hold true but approximately holds true according to QD. The crosstalking was introduced to represent the relationship of two sets of data, {*x*_i_, *i* = 1, 2, …, *N*} and {*y*_i_, *i* = 1, 2, …, *N*}, if the power law does not hold true. There are then three situations. For the first situation, the power law exactly holds true so that the SSE is exactly conserved and the self-similarity exactly holds true. For the second situation, the power law does not exactly hold true but approximately holds true according to QD so that the SSE is approximately conserved and the crosstalking was self-similar. For the third situation, the power law does not hold true evenly according to QD so that the SSE is not conserved and the crosstalking was not self-similar.

The calculation of the self-similarity of the HRV parameters in different processes showed that the association between trait mindfulness and the HRV parameters did not emerge at either the absolute level or the difference-change level; however, this association emerged at the crosstalking level. These results are consistent with a preliminary study investigating the physiological mechanisms of mindfulness conducted by Sun et al. (2018). The crosstalking of HRV parameters in different processes indicated that the more mindful people are, the more likely they are to maintain ANS function homeostasis. Moreover, we used as many HRV parameters as possible to represent ANS function because organisms may react to continuously changing internal and external contexts holistically rather than as a separate function represented by one or two parameters. Notably, although we emphasize the synthetical and interactive functions represented by different measures, we do not overlook the functions represented by a single specific measure.

Our study showed that the MAAS score was not significantly correlated with the HRV parameters in the calming phase. This finding is consistent with the results reported by Jäger (2016), who found that the MAAS score was not significantly associated with SDNN, LF-HRV or VLF-HRV in a resting state within a sample of 106 undergraduate students. Furthermore, we did not find any significant correlations between the MAAS score and the HRV parameters in the calming phase or the other three phases. Some studies have revealed that trait mindfulness was associated with HRV parameters, especially HF-HRV, which is indicative of PNS activity, during the recovery phase. For example, Garland (2011) found that higher trait mindfulness was significantly associated with greater HF-HRV recovery from stress-primed alcohol cues among 58 alcohol-dependent inpatients. Fogarty et al. (2015) found that participants who were more mindful showed greater HF-HRV during an emotional writing task, followed by superior recovery. Trait mindfulness may facilitate more adaptive self-regulation after stressful situations in the recovery phase. However, our results did not support this conclusion. This discrepancy might be related to the nature of the tasks assigned. Garland (2011) and Fogarty et al. (2015) assigned an emotional task to the participants, while we used a cognitive task. Future research should investigate whether the nature of the tasks led to this difference.

The second part of the findings pertains to the bivariate correlations between the MAAS score and HRV parameters at the difference-change level. Neither the HRV parameters in phase 2–phase 1, indicating mental arithmetic task reactivity, nor the HRV parameters in phase 4–phase 3, indicating mindfulness practice reactivity, were significantly correlated with the MAAS score at the difference-change level. These findings are consistent with those reported by Soer et al. (2015), who also found no significant correlation in the change scores between trait mindfulness and the coherence score after three sessions of coherence training. There is some agreement that trait mindfulness reflects a greater tendency to abide in mindful states over time (Brown et al., 2007). Trait mindfulness is likely associated with HRV parameters in different states or the difference-change level of HRV parameters. However, from the perspective of process philosophy and the Weber–Fechner Law, it is better to understand the terms “over time” and/or “across situations” in the context fold level of physiological reactivity. After applying the new self-similarity algorithm, our results revealed that the association between trait mindfulness and the HRV parameters exists at the crosstalking level of the HRV parameters but not at the difference-change or absolute levels of HRV parameters. Because the algorithm of the self-similarity of HRV indicates ANS function homeostasis and our findings revealed an association between trait mindfulness and the self-similarity of the HRV parameters, we can cautiously infer that trait mindfulness could help keep different biological functions in homeostasis in individuals facing a variety of challenges. Further investigation is needed to clarify the underlying biological mechanism of the adaptive function of trait mindfulness.

In summary, the findings of the current study showed that trait mindfulness is associated with the indexes of the self-similarity of HRV but was not significantly correlated with HRV at the absolute level or the difference-change level. These results suggest that mindful individuals who tend to be acutely aware of their states in the moment can flexibly adjust their psychological and physiological reactivity and reactions according to internal and external signals, resulting in FSH maintenance.

The current study failed to identify associations between trait mindfulness and the HRV parameters at the absolute level and the difference-change level. There are at least two explanations for these findings. First, the self-rating on a global measure of mindfulness as indicated by the MAAS score could be unrelated to how mindful that individual could be in a specific state. Self-ratings of how mindful one is while coping with a situation may better capture how mindful individuals are during the given situation. Hence, pairing state measures of mindfulness with state measures of ANS responses might be more effective in future research. Second, inspired by our findings mentioned above, the association between trait mindfulness and the HRV parameters may unfold at the crosstalking level in different processes. The findings suggest that individuals with high trait mindfulness show flexible regulation of the ANS, which is a marker of adaptation.

There are several limitations to the current study. First, the sample consisted of undergraduate students from a Chinese university. Future research is needed to establish whether these findings can be generalized to other populations. Second, the mental arithmetic task in phase 2 was a cognitive task that represented a break from daily life and could not induce complex emotions. An emotional task should be established to investigate whether the results obtained in the current study could be repeated.

## Conclusion

The present study suggests that the experience of mindfulness in daily life could increase the likelihood of maintaining ANS function homeostasis as indicated by the self-similarity of HRV. As proposed by Liu C.Y. et al., 2017; Liu T.C. et al., 2017; Liu et al., 2018, health is the ability to keep one’s functions self-similar; our findings further suggest that being aware of one’s present experiences as they unfold, i.e., trait mindfulness, may prevent biological processes that could damage health. In summary, the homeostasis demonstrated by the self-similarity of the HRV parameters may be a mechanism explaining why trait mindfulness has adaptive merits. The exact mechanism is worth investigating in-depth in future studies.

## Author Contributions

SS, JP, and MH made important contributions in designing the experiments and writing this paper. SS and CH contributed with data collection and data processing. SS, CL, and JP helped to analyze the data. CL made substantial contributions in revising the manuscript critically for content relating to self-similarity algorithm, and was responsible for the algorithm. All authors made substantial contributions to the manuscript and revised the manuscript for several times. All authors read and approved the submitted version.

## Conflict of Interest Statement

The authors declare that the research was conducted in the absence of any commercial or financial relationships that could be construed as a potential conflict of interest.

## Footnotes

1 WeChat is one of the most popular social media platforms in China.
